# Investigating technology development in the energy sector and its implications for Indonesia

**DOI:** 10.1016/j.heliyon.2024.e27645

**Published:** 2024-03-06

**Authors:** Maxensius Tri Sambodo, Mesnan Silalahi, Nur Firdaus

**Affiliations:** a Center for Behavioural and Circular Economics Research, Indonesia National Research and Innovation Agency (BRIN), Indonesia; b Universitas Nasional, Graduate School of Political Science, Indonesia; c Gadjah Mada University, Faculty of Economics and Business, Jakarta, Indonesia; d Graduate School of Global Environmental Studies, Kyoto University, Kyoto, Japan

**Keywords:** Technology development and innovations, Disruptive technology, Renewable energy, Energy efficiency, Low carbon, Indonesia

## Abstract

Innovations for a low-carbon economy and carbon neutrality are the focal points of technology development in the energy sector. This paper aims to investigate the progress of technology and advancements in the energy sector and the implications for Indonesia via two routes, viz., renewable energy and energy efficiency. The methodology employed in this research is divided into two parts. Firstly, an extensive literature review was conducted to identify prevalent research trends within the energy sector. Second, a case study method was performed to gain a comprehensive understanding of energy transformation within the electricity and steel sectors. The study presents two key findings. First, the energy sector has experienced significant technological disruption, providing the opportunity for Indonesia to transition towards a low-carbon development. This includes smart grids, energy-intensive industries, electric vehicles, and storage (pumped storage and battery). Second, lessons from the steel industry show that the technology selection is sensitive to parent company, and this is not easily captured in literature studies. Indonesian has gained many benefits from the global technological disruption, but this is not sufficient without developing changes in support system and consumer behavior.

## 1 Introduction

To address the enormous challenge and opportunities presented by climate change, research and technology development have advanced to transform our energy systems, and work to overcome market barriers in renewable power and energy efficiency. With regards to technology development and innovations (TDI), notable advancements have been achieved in recent years. These enable us to produce clean and environmentally friendly energy cost-effectively. However, there are still many challenges in the energy sector that need to be solved through innovations. Some of these challenges include the promoting technology to extend sustainability of fossil fuels and financial innovation in solving the stranded assets of coal-based energy infrastructure. At the country level, this translates into national imperative to develop a strategic plan to tackle the challenges. This entails acquiring knowledge regarding the current state of the research and innovations towards low carbon development.

Most contemporary social, economic, and environmental predicaments necessitate creative solutions grounded in innovation and technological advances [1]. The concept of creative destruction to denote innovation that disrupts the existing order, leading to the creation of a new order [2]. Disruptive technology (DT) is a kind of unexpected TDI that brings rapid transformation in both benefits and production costs [3]. Disruptive technology has challenged existing technology, making it obsolete, and if the firms fail to innovate, the new entrants will sweep the incumbent out of the business [4]. Furthermore, compared to established technology, disruptive technologies are more straightforward, cheaper, reliable, and convenient [5,6]. Disrupted innovation is related to the emergence of novel ideas and technologies resulting in advanced goods and services that are readily available and economically accessible for a broader consumer base, essentially altering conventional business structures. In relation to low-carbon innovations, this provides benefits to customers through value propositions and has the potential to revolutionize energy markets and reduce emissions at the same time [7]. Such a progress has become the central theme of technology development in the energy sector. As mentioned in the Global Innovation Index 2018, the direction of innovations in the energy sector aims to boost innovation in climate-friendly green technology while fulfilling a rapid increase in energy demand [8], along with the projected global energy demand to increase by 30% in 2040, requiring a higher climate-friendly energy supply. DT in the energy sector will drive the electricity industry through innovations in the renewable energy, energy efficiency, energy conservation and storage [9] that will contribute to the emission reductions [10].

Identifying DT in the energy sector needs to consider current innovation agendas. The World Energy Council highlights advancements in electric storage and renewable energy that determines the pace and the magnitude of the energy transition driven by the replacement from wind and solar PV to replace hydrocarbons [11]. Improvements in battery technology will revolutionize the transportation sector through the electric vehicle (EV). These advancements, including other technological innovations (e.g., energy storage, plastic recycling, LED light efficiency, carbon capture and storage/CCS, and hydrogen) will shape the sustainability agenda in relation to the energy transition [12]. In addition, with the rise of disruptive blockchain technology and the advancement in the demand response system in the energy sector, there are three fundamental principles used to determine future energy systems, such as decarbonization, decentralization, and digitalization [13].

Systematic efforts to reduce carbon emissions have been carried out by the Indonesian government. Under the National Action Plan for Reducing Greenhouse Gases 2011, there were 26 action plans in the energy and transportation sectors have been set, such as rapid mass transportation (MRT), double track for train, Jakarta monorail, energy management mandatory, efficiency on household equipment, and energy management provision for new and renewable energy. Further, under the documents of enhance national determined contribution (NDC) 2022 and Long-Term Strategy on Low Carbon and Climate Resilient Development 2050, energy sector has an important role to play in achieving greenhouse gases (GHGs) reduction target.

DT provides a greater opportunity for Indonesia to reduce greenhouse gas emissions by 31.89% under unconditional conditions (relying on national strengths) and 43.2% with conditional conditions (international cooperation) by 2030 (Table 1). CO2 emissions from the energy sector were the second-largest contributor after the forestry sector in 2010 (Table 1). However, it is predicted that the energy sector will emerge as the main contributor of emissions in 2030. This implies that emissions reduction from the energy sector will significantly impact the national target. In the proposed enhanced NDC in the energy sector, the Indonesian government has identified six measures for mitigation [14], namely: enhancing efficiency in the final energy consumption by improving device and energy system efficiency; adopting clean coal technology in power plants; expanding the deployment of renewable energy; utilizing biofuels derived from palm oil in the transportation sector; improving access to gas pipelines for households and commercial sectors; and increasing the number of compressed natural gas stations.

**Table 1:** National determined contribution the Republic of Indonesia.

No	Sector	GHG Emission Level 2010*	GHG Emission Level 2030			GHG Emission Reduction				Annual Average Growth BAU (2010–2030)	Average Growth 2000–2012
		MTon CO2e	Mton CO2e			Mton CO2e		% of total BaU			
			BaU	CM1	CM2	CM1	CM2	CM1	CM2		
1	Energy*	453	1669	1311	1223	358	446	12.5	15.5	6.7	4.5
2	Waste	88	296	256	256	40	43.5	1.4	1.5	6.3	4.0
3	IPPU	36	70	63	63	7	9	0.2	0.3	3.4	0.1
4	Agriculture	111	120	110	108	10	12	0.3	0.4	0.4	1.3
5	Forestry**	647	714	214	−15	500	729	17.4	25.4	0.5	2.7
	Total	1334	2869	1632	1787	915	1240	31.89	43.2	3.9	3.2

Note: BaU (Business as Usual); CM1 (unconditional mitigation scenario); CM2 (conditional mitigation scenario); * including fugitive; ** forestry and other land uses (FOLU) – including emission from estate and timber plantations.

The aim of this study is to investigate the routes of TDI within the energy-intensive sectors namely the electricity sector (generation and distribution) and the steel industry with respect to achieving a low carbon development in Indonesia. This approach provides a broader perspective to open a window of opportunity for Indonesia to utilize TDI and DT to achieve the NDC targets in the energy sector. We explore the direction of TDI in the energy sector and its implications for Indonesia by applying two strategies. First, we reviewed previous studies to analyze the trends in the energy-related sectors. Second, we analysed the practices of TDI in the selected case studies such as the power sector and steel industry. The first strategy can help in mapping technology development and linkages in a global context. Meanwhile, the second strategy will help determine the sector's position in the global context. By applying the two strategies, the policy context that needs to be prepared can be found to encourage development and innovation of technology, a technological leadership position at the industrial level.

This study finds that TDI needs to be seen in the context of a system that requires a response from both the supply and demand sides. Local resources, capabilities, demand conditions, and policy context will determine the scale and speed of disruption. Furthermore, this research also finds that technology choices at the company level cannot necessarily be captured in global literature observations, although some technology characteristics can be mapped. This illustrates that technological advances have built new configurations at the company level with unique specifications. TDI has a profound implication to energy sector in Indonesia namely in the power transmission and distribution through the disruptive smart-grid technology, low-carbon and energy efficient steel industry and the energy storage through batteries. This paper emphasizes the necessity for Indonesia to facilitate a shift in its energy sector, from a fossil-fuel based energy system to a low-carbon energy system, whereby TDI and DT need to be placed strategically in the context of promoting energy security.

Country-level studies have tried to address DT with a more specific scope, such as heat decarbonization in the United Kingdom [15]. In addressing DT, they pointed out three goals that need to be addressed by policymakers [15]. First, the government can reduce uncertainty by developing research and development efforts. This will assist in gathering the necessary information to get out of the bounded rationality trap. Second, the government can focus on ‘low regrets options initially’ and prioritize the scenarios that will yield the most significant effects on the energy transition. Third, the government needs to acknowledge the inevitability of uncertainty during the energy transition process. Thus, government needs to possess ‘future toolkit’ in order to enhance the implementation of adaptive governance. Thus, the novelty, as well as the strength, of this study is in the approach to in-depth investigation of the TDI and DT pathways comprehensively in energy intensive sector in Indonesia using mixed methods by juxtaposing elaborated Scientometrics of global research publications with enhanced rigor using content analysis (co-word analysis with visualization and topic modeling), to better understand the current status. However, the study is limited by the minimal numbers of experts allocated in the in-depth interview and sector coverage. Further, the potential impact of carbon tax is not discussed at the case study. The remainder of this paper proceeds as follows. The next section explores the datasets and research methods content analysis using co-word analysis and topic modelling, and the case studies. Section 3 presents the results and discussion of selected case studies. This section begins with exploration of the main issues in the energy transformation and the affected industries. Section 4 concludes the paper and discusses policy implications.

## 2 Materials and methods

### 2.1 Research Framework

**Fig. 1:**
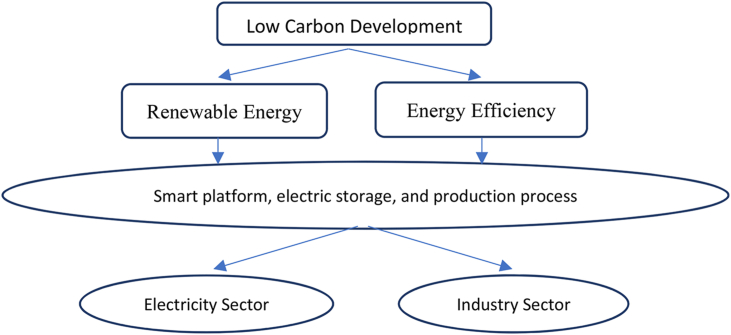
Research framework.

DT can be promoted by two ‘engines’—renewable energy and energy efficiency (Fig. 1). These engines are fuelled by smart platform, electric storage and market design. Reducing greenhouse gas emissions in 2050 will be through six technological pathways, namely renewable energy, energy efficiency, electrification, hydrogen, CCS, and bioenergy with CCS [16]. Half of the 36.9 GtCO2 that is expected to be reduced will be attributed to the equal contributions of renewable energy and energy efficiency. This study analyzes what kind of facilitation can be provided by disruption in terms of smart platforms, power generation, electricity storage, market design, and business model, helping to achieve this goal. Two sectors significantly contributing to GHGs in the energy sector in Indonesia, namely electricity and steel industry were selected as case studies.

### 2.2 Systematic literature review (SLR)

Renewable energy and energy efficiency have emerged as two fundamental aspects of achieving low-carbon development, yet it is crucial to conduct a comprehensive analysis through a systematic literature review. A systematic literature review can be performed to assess research trends and synthesize the findings [17] with a recent and well-organized depiction of the literature within a particular field of study. To this end, two methods of the content analysis (co-word analysis and topic modelling) in the scientometric study are applied to acquire a view of the global research in selected case studies. Four datasets related to disruptive technology in the energy sector was collected from the Scopus© database using the following search strings.

• Disruptive energy transformation: TITLE-ABS-KEY (“energy” AND “transformation” AND “low carbon”) AND TITLE-ABS-KEY (“disrupt*” OR “technolog*” OR “innovation?”) AND PUBYEAR >2000 (960 records).
• Smart grid: KEY (“smart grid*" OR smartgrid* AND technolog* AND (roadmap OR direction* OR disrup* OR future OR advancem* OR develop*)) AND PUBYEAR >2000 (931 records)
• Low carbon power generation: TITLE-ABS-KEY (decarbon* OR “low carbon” OR “emission reduction”) AND TITLE-ABS-KEY ((“technolog*" OR “innovation?”) AND “power plant*") AND PUBYEAR >2000 (2264 records).
• Low carbon iron and steel industry: TITLE-ABS-KEY (“steel industry” OR “steelmaking” OR “ironmaking”) AND TITLE-ABS-KEY (“energy management” OR “energy efficiency” OR “energy conservation” OR “low carbon” OR “decarbon*” OR “emission reduction”) AND PUBYEAR >2000 (2853 records).

The author index keywords, and the abstracts of the Scopus dataset were presented. Based on these datasets, we performed content analysis and bibliometric methods using VOSViewer to show issues related to disruptive technology in the energy sector. Co-word analysis [18] explores the interaction between keywords across a population of texts and the depiction of such co-occurrences by graphical method. In addition, a topic modeling analysis [19] is carried out for in-depth analysis because of the much richer developments in the iron and steel industry. Topic modeling makes it possible to automatically identify meaningful patterns and correlations between words that frequently appear together in a corpus of documents [20]. On the basis of a content analysis, we carried out a qualitative meta-analysis in order to gain a thorough understanding of the results regarding a specific subject examined in various studies [21]. By combining the findings of different qualitative studies, a qualitative meta-analysis can strengthen the validity of conclusions.

### 2.3 Qualitative analysis

We adopted a case-study method in the qualitative study. We used primary sources from interviews with selected stakeholders (e.g., academics, policymakers, professionals), focus group discussions (FGDs), and field surveys and analysed such data qualitatively. We interviewed experts from the government, Indonesian Electrical Power Society (MKI), Indonesia National Research and Innovation Agency/BRIN (formerly Indonesian Institute of Sciences/LIPI), a state-owned electricity company PT PLN (Persero) and its subsidiaries (e.g., PT Indonesia Power and PT Pembangkitan Jawa-Bali (PJB)) as power generator and distributor companies, PT LEN (electronic equipment producer), and PT Krakatau Posco (steel producer). These experts are actors involved in policymaking, developing renewable energy technologies (i.e., solar PV and battery storage), and technology utilization. Secondary sources, such as documents and reports, are also used to complement the analysis.

We adopted a multi-case study method to investigate the implications of disruptive technologies on the energy sector in Indonesia. This method enables to explore the reasons behind the emergence of disruptive technologies in the energy sector and their impact on Indonesia's energy sector. In this context, we documented the reactions of stakeholders involved in disruptive energy technologies. We employed an integrated analysis of energy transformation dynamics based on empirical observations. This can be identified from business performance data and direct interactions with stakeholders. We conducted an investigation into the power sector and the steel industry. We begin with the idea of carbon emission reduction efforts for the power generation sector in which the power system, hitherto, is dominated by steam and coal power. To this, we then explored how the existing power will develop in the future, considering the emergence of “smart” and low-carbon technologies.

## 3 Results and discussion

**Fig. 2:**
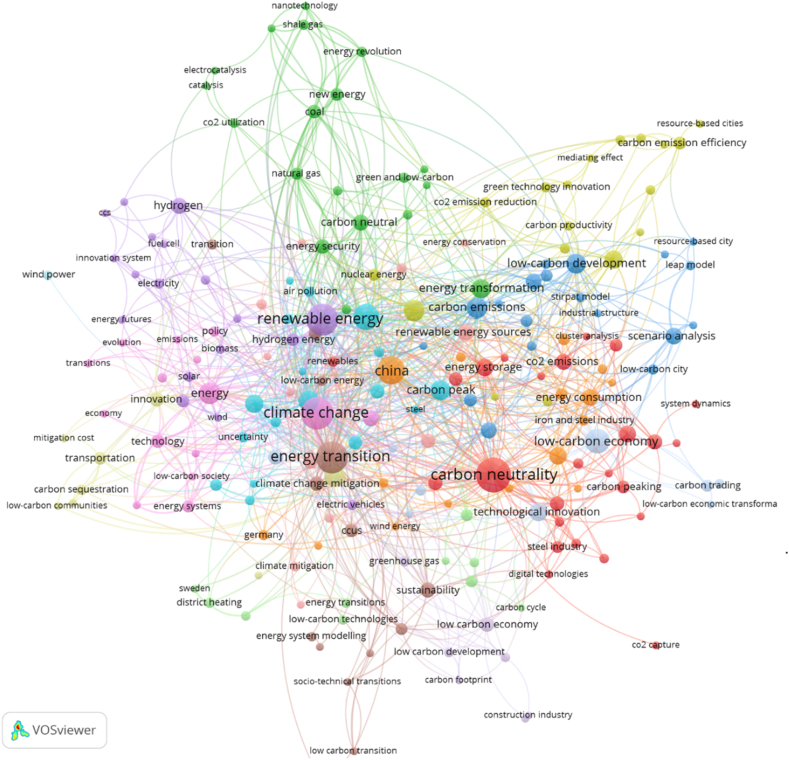
The mapping of co-word analysis of the global disruptive energy transformation.

Over the last ten years, driven by the disruptive technologies and innovations, the generating cost of electricity from renewable energy has drastically declined. This implies that renewable energy, mainly driven by wind and solar PV, has reached the mainstream market; hence, it is projected that renewable energy will be the future technology. Meanwhile, there exist various avenues through which energy efficiency can enhance the global energy system, including strengthening energy security, curbing energy consumption, and helping the environment [22,23]. Fig. 2 shows the relationship between key concepts in global research regarding disruptive technology in the energy sector. Several crucial topics related to disruptive energy transformation include energy efficiency, energy utilization, digital transformation, and sustainable development. In relation this, the most affected sectors include the iron and steel industry, power and electricity industry, transportation (electric vehicles), and construction industry.

### 3.1 Electricity sector

In relation to energy efficiency, a ‘smart platform’ covering smart meters, grids, and smart electronic devices is essential [23]. Moreover, digitalization has a significant impact on energy efficiency, and it creates an opportunity for integrating efficiency with renewable energy to reach the least cost [22]. Digitalization refers to efforts to promote efficiency by adopting new technology such as e-payment and smart meters or smart grids. According to IEA [24], as part of a smart platform, a smart grid refers to an electricity network that utilize digital and other cutting-edge technologies in order to monitor and manage the transmission of electricity from all sources of power generation, with the purpose of meeting the varying electricity demands of end-users. The objective behind the promotion of smart grids is to minimize costs, mitigate environmental repercussions, and optimize the system reliability, resilience, and stability.

However, collaboration among generators, grid operators, end-users, and electricity market stakeholder are critical for the efficient functioning of smart grids. How far smart grids can be deeply implemented depends on local commercial attractiveness, technology capability, regulatory development, and investment framework [24]. Besides, information regarding electricity demand, the deployment of electric vehicle (EV)and peak demand in relation to EV deployment, as well as the potential for demand response, future electric use in buildings, and the implementation of advanced metering infrastructure drive the successful of smart grids [24]. In addition, several issues need to be studied, such as the complexity of power system in ensuring interoperability among the players involved, cyber security, a business model in anticipating the rise of ‘prosumer’, strong and smart grid transmission with modern high voltage direct current (HVDC) technologies, and the response of distribution system operator (lead to one player or many players). Generally speaking, the policy arena in promoting smart grids is to promote renewable energy in the generating sector, develop regulations to ensure fairness in sharing risks, costs, and benefits among players, develop standards, and support R&D.

In response to the issue of climate change, researchers worldwide are working towards developing enabling technologies to assure reliable and robust low-carbon electricity systems such as renewables (solar power and wind power), nuclear power, fossil carbon capture and sequestration (fossil-CCS), Integrated Gasification Combined Cycle (IGCC) plants, waste heat recovery, low-cost electricity storage, and smart power grids. Fossil generation will need to be outfitted with CCS technology if it is to continue being a part of the future electrical system. Coal power plants could potentially remain a substantial component of the energy grid through the utilization of CCS technology. However, the implications for future scenarios are significant when it comes to understanding the complexity of existing fossil fuel power plant decarbonization as the integration of fossil fuel power decarbonization in a future energy system is subject to a multitude of economic and policy uncertainties [25]. The primary obstacle to putting into practice fossil CCS is that there are still insufficient energy incentives for reducing emissions from power generation. However, in scenarios with stringent climate policy role, CCS technology is a prominent option within the strategies aimed at mitigating emissions, particularly in situations that entail substantial reductions in GHG emissions [26].

Research on energy conservation pursues the development of systems that are an effective and economic CO2 capture technology pathway and has the reduction of CO2 emissions as one of its main objectives [27]. IGCC plant equipped with CO2 capture is a pre-combustion process where hydrocarbon fuels are converted to a mixture of CO2 and hydrogen, which then be used as fuel for power generation. IGCC is a power generation technology wherein solid feedstock is partially oxidized to produce syngas. This syngas is then utilized to power high-efficiency gas turbines with the waste heat being recovered and converted to generate electricity. The integration of the gas turbine integration enables substantial power generation in which it is essential to optimize the operating conditions of the gasifier in order to ensure the performance of the integrated system [28]. Post-combustion capture is applicable to conventional pulverized coal boilers and natural gas combined cycle (NGCC) plants. The utilization of oxy-fuel combustion enables the possibility of capturing large-scale CO2 from a coal-fired power plant, thereby serving as an efficient means of to reducing CO2 emission and achieving carbon neutrality [29]. However, the disposal of the ultra-low volatile carbon-based fuels produced by the coal chemical industry presents a challenge in terms of effectiveness, and a potential solution could be the co-firing [30]. Hydrogen is poised to emerge an increasingly disruptive energy vector, presenting immense prospects for fostering the utilization of intermittent renewable energies.

**Fig. 3:**
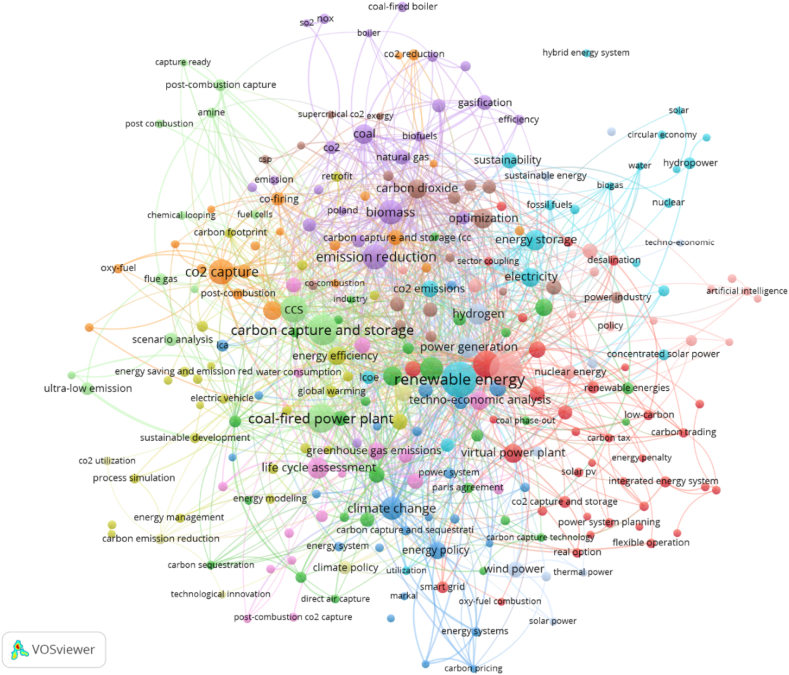
The mapping of global research on low carbon power generation.

Fig. 3 visualizes the mapping of the global research on low-carbon technologies in power generation indexed in the Scopus© database. The green-colour cluster is topics on fossil-based electricity generation dominated by clean coal-fired power generation. The low-carbon scenario is pursued through carbon capture and storage (CCS), waste heat recovery, hydrogen production, methane capture, fuel cells with hydrogen, bioenergy, biofuels, energy conversion, and combined heat and power. Other important terms are: “renewable energy”, biomass, cogeneration, co-firing, gasification, hydrogen, and “coal phase out”.

Many companies have made efforts to adopt new technologies and this provided information in terms of technology capability and its applications. In Indonesia, securing the primary energy supply is critical to providing sufficient and affordable electricity. In line with this, adequate funding is necessary to support business development and operating activities, including investment in the generation, the transmission and distribution network, particularly to strive towards achieving a more sustainable energy system. Besides, decarbonization requiring a large share of renewable energy technologies is no longer centralized in which such technologies can be spread over several areas on a smaller scale. Yet, infrastructure development is the key to achieving the transition goal, so a large investment is needed. In addition, the deployment of renewable energy technologies is less effective without being supported by energy storage development concerning the intermittent problem.

Furthermore, it is worth noting that collaboration with multiple parties, such as Independent Power Producer (IPP) and financial institutions, is critical to accelerating decarbonization. In this regard, it should be realized that the capacity of PLN and its subsidiaries is limited, mainly to scale up green energy projects and digitize electricity businesses. Thus, the challenge is attracting other parties to participate in the electricity generation industry facing a low-carbon world. From the demand side, renewable energy has been promoted by multinational companies such as Apple, Microsoft, Danone, H&M, and L'Oreal, planning to use 100% renewable energy for their business. The mechanism can work under renewable power wheeling (RPW), which directly transfers power from renewable energy sources to the company's operational facility. For example, L'Oreal that is located in Cikarang-Bekasi, West Java, has bought electricity from hydropower in Kracak, Sukabumi, that owned by the Indonesia Power since 2013. Encouraging disruption from the demand side needs to continue, either through regulatory channels or corporate awareness through environmental, social, governance (ESG) commitments. However, it is critical to consider the risks and share them fairly under the power purchase agreement and this is part of the enabling environment where a good governance, risk, and compliance (GRC) framework needs to be built. Indonesia has been experiencing several business models, such as take-or-pay, balance business engagement, risk-sharing, delivery-or-pay, and take-and-pay. Therefore, MKI suggests that GOI establish a new agency (Regulator Agency/Regulation Enforcement Body) regarding the governance system. This agency should be an independent body that can objectively determine the tariff, protect consumer needs, monitor, and control the implementation of regulations, and provide rewards and punishment. Also, PLN needs to reduce the impact of disruptive technology through excellence in absorptive capacity [31]. The current shift in energy focus revolves around innovative designs that creatively blend technologies and explore unique business approaches. It's evident that innovative strategies from businesses, communities, and governments can collectively bring the necessary disruption to address our pressing national and global challenges [32].

Moreover, because most renewable energy, such as geothermal projects located in remote areas, promoting renewable energy needs to be balanced with creating demand near the location. In this regard, providing transmissions and distribution infrastructure should be encouraged. Yet, the government needs to expand insurance schemes, promote pricing incentives, and facilitate funds in response to the risks associated with exploiting renewable energy. The support system developments may include geothermal fund facilities, solar PV and solar rooftop leases, and a business model for distributed solar PV. Therefore, it is recommended that governments and international financing agencies adopt a strategy that integrate private investments into electricity generation.

**Fig. 4:**
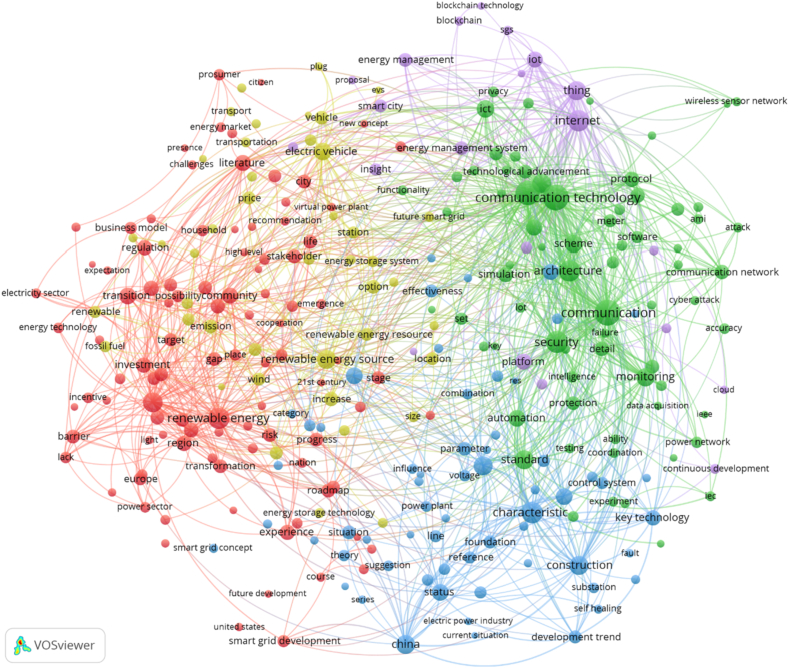
Clusters of topics in global research related to future technology in smart grids.

Electricity grids become smarter by digitalizing and integrating communication and networking capabilities into the grid. Then the price signals are used by the smart grids to regulate electricity use. There are many issues related to the goal of the development of a self-healing smart grid system. Fig. 4 shows important global issues in the development of smart grids. The cluster in green colour is related to communication technology which has linkages with terms like standard, protocol, architecture, smart meter, application, and security. There are issues such as policy, regulation, roadmap, transformation, transition, business model, and integrating renewable energy, grid-to-vehicle, energy storage, blockchain technology, and internet of things (IoT). According to IEA [33], across the energy sector, future challenges and opportunities are outgrowing current policy, regulatory and governance processes and structures. The nature of industry 4.0 in the electricity sector will be the combination of renewable energy, smart grid (micro-grid and hybrid), electric vehicles, energy storage, internet of things, customer-centric (personalized), integration of control system and measurement. This new trajectory needs a well-planned transition and a new mindset from decision-makers. Developing an infrastructure that provides more benefits from the technology disruption is vital. The future situation will transform a traditional grid into a dynamic infrastructure with the smart grid. This transformation would develop a transmission and distribution system compatible with the distributed energy resources including integrating SCADA (Supervisory Control and Data Acquisition) systems, upgrade the controlling operating system, develop infrastructure for electric vehicle charging, upgrade the conventional sensor on the telecommunication network, and prepare the customer-centric approach by utilizing big data and artificial intelligence.

Many developing countries are increasingly recognizing the importance of smart grids as a means to modernize their energy infrastructure, increase energy efficiency, and integrate renewable energy sources into their grids, especially related to promoting smart cities [34]. Regarding the country-level issues and challenges of smart grid development in Indonesia, our analysis shows signals of disruptive technology in Indonesian research on smart grids. A shift occurs in research topics over a relatively short time related to a smart grid. Research topics on the smart grid in Indonesia were identified as growing in line with the global trend toward micro-grids and the internet of things (IoT). This trend has been regarded as the pillar of industry 4.0. Related to this are keywords like energy efficiency and distributed generation, smart micro-grids, smart metering, energy management systems, demand response, intelligent buildings, smart homes, and wireless sensor networks; internet protocols, cyber security, and cryptography; forecasting, monitoring; big data, and deep learning; EVs, and battery storage. This highlights the significance of the smart grid in the context of EVs, smart cities, intelligent buildings, and smart homes. Network security becomes a crucial concern in this scenario. Future research and development are going towards the smooth integration of long-distance efficient electric power transmission networks based on distributed generations of various renewable energies with the end goal to develop a self-healing smart grid system. Furthermore, energy policy for sustainable development of rural electrification, whereas mainly hybrid systems based on renewable energy, the economic and social appropriation, and electricity demands in agriculture will add to the complexity.

An expert from PLN said that three significant issues affecting the future of the electricity business in Indonesia are decarbonization, digitalization, and decentralization. Decarbonization is the most critical issue while digitalization requires recognition of the importance of building a smart grid infrastructure as early as possible in tapping the full benefit of the new technologies. This is also related to the trend of decentralization in the energy sector. In addition, smart grids also play a vital role in energy efficiency and become a key driver in transition to renewable energy. Smart grid development is a government program and has become part of the 2020–2024 Medium Term Development Plan (RPJMN), and together with PLN will develop smart grids in stages. To this end, PLN establishes a smart grid working group and starts from the existing PLC (Power Line Communication) and SCADA (Supervisory Control and Data Acquisition) implementation. PLN has collaborated with China to build Automatic Metering Infrastructure (AMI). Its deployment can increase efficiency (up to 55%) and productivity while increasing the quality of services to its customers. It is projected that the implementation of smart meters will completed for a total of 1,217,256 customers by the end of 2023, encompassing several regions including East Java, Central Java, West Java, Jakarta, Banten, Bali, Medan and Makassar [35]. Through this infrastructure, smart metering implementation becomes possible. The optimal approach is designed with the ability of automatic meter reading. A smart grid is a complex automation and data exchange system with service-oriented energy management architecture. More than 100 IEC standards have been identified as relevant to the smart grid related to service-oriented architecture, energy management, power utility automation, distribution management, security, data exchange for meter reading, tariff and load control, and functional safety of the systems. The Indonesian standards which have been developed to support smart grid implementation are 18 SNI (Indonesia National Standard) in a photovoltaic system, 7 SNI in the battery system, 7 SNI in control and energy management systems, 7 SNI in generator system, and 9 SNI micro-hydro system [36]. This implies that Indonesia needs innovations to optimize grid operations and develop more national standards in the smart grid sector to ensure the smooth implementation of smart grid technology in Indonesia.

Furthermore, PLN has implemented SCADA technology to monitor and control electronic devices or power systems from long distances in real-time and computer based. SCADA can acquire information from the main transformer or distribution transformer. Then it processes information and responds by analysing data. The study showed that SCADA could reduce disturbances and improve the reliability index of power supplies such as SAIDI (system average interruption duration index), SAIFI (system average interruption frequency index), and CADI (customer average interruption duration index). The current SCADA system is the first generation or wire-based system, and it needs to be wireless in the future also, the capacity of the transformer to make self-adjustment needs to be improved. The application of SCADA in the distribution system at Palu City, Central Sulawesi, for instance, showed that the reliability parameters such as SAIDI, SAIFI, and CAIDI improved by 41.49%, 32.31%, and 13.55%, respectively [37]. The advanced SCADA system can push the system to develop a self-healing system for any problems. It may need five to ten years for PLN to develop an advanced SCADA system. SCADA can improve system reliability and efficiency and select the least cost energy sources. PLN has prepared a roadmap to the smart grid for the future. PLN has also prepared the transition from SCADA to smart grid which will require regular analysis and testing of its performance. For example, the smart grid has been implemented in the industrial zone Surya Cipta Sarana, Karawang, West Java. PLN collaborates with several agencies in this pilot project, such as the Ministry of Energy and Mineral Resources (MEMR) and the New Energy and Industrial Development Organization (NEDO). PLN argues that smart grids need to be developed and implemented in regions with high penetration of renewable energy and small and remote islands. Concerning this, Load Frequency Control (LFC) and Automatic Generation Control (AGC) have been implemented in the Java-Bali system. Currently, 98 generators in Java-Bali out of 130 units have AGC.

The application of smart platform technology (smart grid) and electricity storage, at the implementation level, can be challenging. The technology lock-in situation and funding constraints mean that up-grading technology needs to be carried out in stages. This is because the adoption of a new technology requires adjustments to other components in the system. When the system design is fragmented, it certainly requires good coordination from all actors to build a low switching costs and transaction costs. The role of regulators is very important in facilitating the process of adopting new technologies, including in determining product standards.

The development and utilization of renewable energy sources, such as solar and wind energy, are greatly affected by technological disruption in energy storage as it enables the balancing of energy supply and demand, as well as the reduction of fossil energy use. Several technologies that have experienced disruption namely: lithium-ion battery, natrium-ion battery, flow batteries, super-capacitor, and wireless charging. Battery is a disruptive component in the implementation of renewable energy technologies. The technological development in lithium batteries includes three components: materials for the cathode, anode, and electrolyte. However, battery technologies should have reliable capacity, security, speed (fast charging), significant power, and sustainability. There is a rapid transition from cobalt and lithium ion-based materials to silicon, manganese, and natrium, magnesium or lithium-based materials in anode components and sulphur, manganese, and nickel-based cathode components. Sulphur-based lithium batteries have the potential for large capacity, while silicon-based batteries have a high fast-charging capability. The safety aspect is determined by the material's composition and the electrolyte component's architectural design.

Global research in developing materials for battery components has shifted rapidly. In this context, Indonesia has positioned itself as a follower in Li-ion batteries’ science and technology development to build an industry in battery manufacturing. Research in silicon-ion batteries in Indonesia has focused on building a manufacturing capacity based on LFP battery (LiFePO4) and developing raw battery materials based on MNC battery (Lithium Nickel Manganese Cobalt Oxide. Its variant, i.e., LiMnFeSiPO4, is designed for both the energy battery application (graphite anode) and the power battery application (for EV) using NaLiZrTiO as anode material. Early in 2019, the Indonesian battery industry entered a new phase with the construction of a hydrometallurgical nickel laterite production facility in Morowali, Central Sulawesi, for the production of nickel manganese cobalt oxide (NMC). The plant is expected to accelerate the production of 2200 electric cars, 711,000 hybrid cars, and 2.1 million electric motorcycles by the year 2025. Although Indonesia does not have the potential for lithium resources, Indonesia is the largest nickel producer globally. For cathode materials, Indonesia is granted enormous nickel resources. Compositions based on cobalt are increasingly being reduced for the reasons of price and sustainability [38] and will be replaced by other materials such as nickel and manganese.

Other technology in energy storage includes pump-up hydropower, thermal energy storage in power plants, superconducting magnetic energy storage, and hydrogen energy storage. At present, the utilization of pump-up hydropower continues to play a critical role in supporting energy transitions in many countries [39]. Yet, because of the different characteristics of each technology, formulating a long-term strategy for developing these technologies is essential, particularly related to the development of technologies. In this case, the availability of unique local potentials should be considered. The energy storage has the potential to mitigate increasing instability with the growing share of intermittent energy sources, reducing the grid's inertia. Hydropower production and storage, for instance, can offer inertia and load-balancing services to the grid. In Cisokan West Java, pumped storage 4 × 260 MW will start to operate in 2024 (520 MW), and a new capacity of 520 MW will be added by 2025, with a total capacity of about 1040 MW. In Matenggeng and Gindule, an unallocated scheme of 900 MW and 1000 MW will be built by 2025. Regardless, pump storage is essential to reduce generating costs at peak load, repair load factor, and improve the capacity factor of steam coal power plants. In the case of Indonesia, a pump storage application was found that can reduce generation costs.

Thus, technological progress in terms of generation, which is driven by renewable energy sources, needs to be supported by promoting of new technologies, for example for transmission, distribution, and metering. In a smart grid platform, opportunities for utilizing renewable energy generation can expand and achieve economies of scale. This result elaborated previous studies that argue the important of smart grid infrastructure [40]. Thus, we argue that technological disruption needs to be placed within a system framework. Then, it is also apparent that technology efforts to increase efficiency are being made in fossil-based technology. This shows that there is a dual track approach to enhance energy efficiency and decrease carbon emissions. The condition of increasingly environmentally friendly energy demand also holds significant function in exerting influence on the supply side. Policy support is needed to build pareto conditions for new technologies to compete with old technologies that are not environmentally friendly, especially in locations where low hanging fruit can be achieved.

### 3.2 Industrial sector: steel industry

The evolving energy transition in steel production has impacts its energy supply, raw materials, and emission reduction strategies. Innovations in production processes have emerged to reduce energy consumption in the iron and steel industry. These innovations encompass optimizing fuel use, reducing, and recycling waste, and adopting more efficient technologies, such as advanced heating systems. The adoption of renewable-powered electrification for steel production offers an opportunity to drive the industry away from its unsustainable reliance on fossil fuels. Incorporating process flexibility through an optimized design of energy management and steel production can offer a route to large-scale green steel production through renewables-enabled electrification [41]. CO2 emissions from the steel industry which accounts for 5–7% of anthropogenic CO2 emission [42], are amongst the most difficult to abate. The steel production encompasses five stages, each offering potential pathways for decarbonization: iron ore preparation, iron making; steelmaking and casting; rolling, and products fabrication. A transformation toward low-carbon steelmaking technologies includes innovative approaches in CCS, hydrogen-based direct reduction (HDR) steelmaking, and electric arc furnace (EAF) steelmaking [43]. Among these decarbonization technologies, Notably, among these decarbonization technologies, EAF stands out as the preferred method for carbon mitigation in the iron and steel industry. For country specific case, seven emission reduction pathways are identified in China [44]: optimizing iron resources, improving system energy efficiency, optimizing energy consumption structure, transforming to low-carbon technologies, enhancing process optimization and reconstruction, enhancing carbon reduction management, and fostering industrial coordination.

Moreover, factors such as the injected coal ratio of blast furnaces, the charge structure of blast furnaces, and interfaces like the iron-steel interface and casting-rolling interface exert a significant impact on energy efficiency, energy consumption and CO2 emission of the iron and steel manufacturing process [45]. The global iron and steel industry's trajectory towards low-carbon development primarily centers on advancing electric furnace processes, hydrogen metallurgy technology, carbon capture, utilization, and storage (CCUS) technology, as well as promoting clean energy utilization. Within the CCUS framework, existing infrastructure can undergo retrofitting, leveraging proven technology to achieve up to a 70% reduction in CO2 emissions for a marginal renewable electricity demand [46].

Researchers classified 22 advanced pathways for saving energy and reducing emissions in the steel industry into three distinct categories [47]. The first category presents an unchanged trend, such as low-temperature sintering, heat recovery from the sinter cooler, double preheating for hot blast stoves, backfire line heat recovery, and tropical headless rolling. The second category indicates an upward trend of technology penetration, at high temperature and pressure, such as hot-rolling process control, top-pressure recovery turbines (TRT), converter negative energy steelmaking, thick-layer sintering, small pellet sintering, high-efficiency coal injection, application of dry vacuum system for molten steel degassing circulation, and heating furnace black body reinforcement radiation energy-saving. The third category presents a downward technology penetration trend, including dry quenching, hot-rolling process control, low-temperature rolling, reducing sintering air leakage rate, blast and dehumidification, and coal moisture control. The converter negative energy steelmaking and coke dry quenching (CDQ) are the most and second most expensive technology whereas the first one has the highest penetration, and the latter is the lowest penetration rate among others [47].

In general, steel is manufactured through two primary pathways, namely via the blast furnace basic oxygen furnace (BF-BOF) and via the electric arc furnace (EAF). About 70% of steel is produced by the BF-BOF route, utilizing raw materials like iron ore, coal, limestone, and recycled steel [48]. Nevertheless, environmental regulations have significantly reduced the application of the BF method. The BF-BOF contributed significantly to global warming higher than the EAF. Recycled steel or scrap steel is the feedstock for EAF's process that uses electricity. The flash ironmaking technology, hitherto, uses hydrogen that can reduce the energy by about 10.8 GJ/t compared to a blast furnace (about 16.7 GJ/t). In the future, hydrogen metallurgy will be a disrupting method of producing steel that is as significant as the electric furnaces, thanks to a growth in the generation of green hydrogen supplied by waste heat or renewable energy [49].

**Fig. 5:**
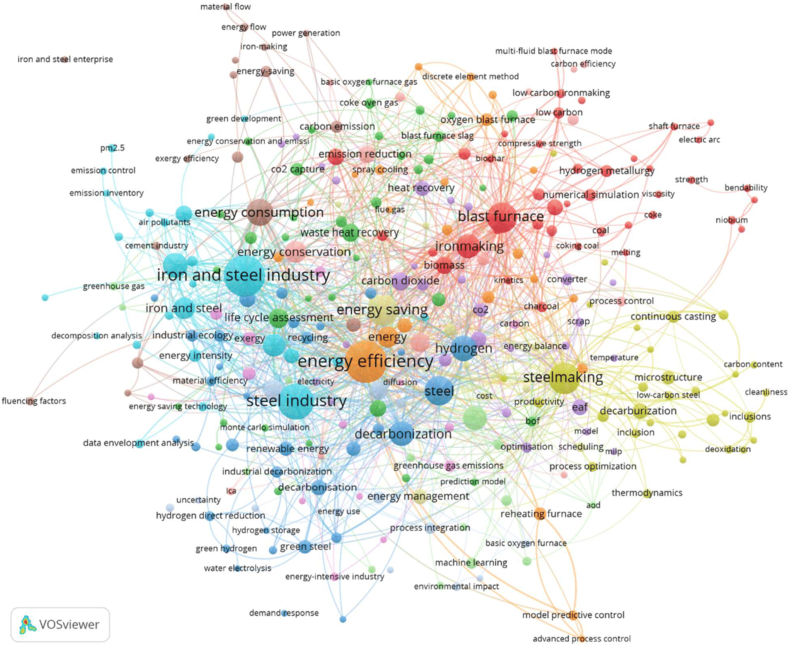
The mapping of co-word analysis on global energy management in the steel industry.

Similarly, the mapping of the co-words (Fig. 5) and its analysis reveals main topics in the iron and steel industry namely related to low carbon development pathways, namely on “energy efficiency”, “decarbonization”, “energy consumption”, “energy conservation, “emission reduction”, and “waste heat recovery”. New trends in the research and development are related to terms such as hydrogen, decarbonization, “life cycle assesment”, “electric arc furnace”, “hydrogen metallurgy”, “hydrogen direct reduction”, “green steel”, electrolysis, “carbon neutrality”, “circular economy”, ccus, “water footprint”, “carbon emission reduction, and “ultra-low emission”.

Other significant terms/phrases are biomass, slag, “heat generation”, steam, pm2.5, “renewable energy”, “green hydrogen”, “exergy efficiency”, “material efficiency”, and “continuous casting”. The term “blast furnace” has a significant importance related to the global research on using biomass in iron making process (Fig. 5). This term correlates with research and development of advanced heating systems and waste heat recovery. Exploration of the inherent potential of recovering energy and re-using heats and materials from waste streams, and high-temperature slag is globally pursued. The steel technology must also deal with low-quality materials such as scrap based. Also, many studies evaluate the long-term potential of energy efficiency alternatives.

Advancements in technology within the iron and steel industry will drive enhancements in energy efficiency. Energy efficiency can be derived from various sources, as illustrated in Fig. 5, which depicts the concept of “energy efficient technology.” However, the extent of energy efficiency achieved depends on factors such as the production process, the types of iron and coal utilized in the steel product mix, energy management during operations, and material efficiency. The transition to decarbonized steel, facilitated by methods like green hydrogen-based iron ore reduction and steelmaking powered by renewable electricity, is poised to disrupt traditional supply chains [50]. Strategies to avoid, capture and store (CCS), or utilize (CCU) CO2 in steel production exist but rely heavily on the availability of renewable electricity.

Steel saves energy over product life cycles in three ways: lightweight, long product life cycle, and recycling. With the new advanced high-strength steel (AHSS), the car's weight can be reduced by 25–39% compared to conventional steel. By lowering the car's weight by 170–270 kg [51], about 3–4.5 tonnes of reduction of greenhouse gases over the vehicle's total life cycle can be achieved. In addition, good quality steel can extend a long product life cycle. In the recycling process, for every 1000 kg of steel scrap made into new steel, more than 1400 kg of iron ore, 740 kg of coal, and 120 kg of limestone can be saved [52].

Table 2 gives the result from topic modeling whereby the topic model (topic size k = 20) is selected and some of the topics are shown with their top terms. The column “Topic Label” shows its reader-friendly interpretation of the top-terms. Overall, this result shows global research which are conducted to reduce the carbon emission in the iron and steel industry by pursuing increased efficiency in the materials and energy consumption, waste heat recycling, and replacing non-fossil (cokes) fuels with green energy. The top-terms intensity analysis shows that carbon capture and storage (CCS) and hydrogen (topic 06), and energy consumption, efficiency, and conservation in iron steel production (topic 12) are the most dominant topic. This is in line with Fan and Friedmann [53] who identify blue hydrogen, carbon-neutral biomass, and CCS appears to have the lowest cost and highest technical maturity. The integration of hydrogen in the decarbonization of the steelmaking processes is regarded as a disruptive one [54]. Topic 15 identifies the significant use of biomass in iron ore reduction process in replacing coal. Major topics on low carbon development (Table 2) are such as electrification in steelmaking using Electric Arc Furnace (topic 04) and heat waste recovery (topic 05) whereby CDQ is a widely adopted technology. Topic 05 identifies exergy analysis much used for informed decision to adopt new technologies.

**Table 2:** Results from Topic Modeling

No	Top Terms	Topic Label
01	control, system, technology, process, systems, steel, energy, design, plant, efficiency, equipment, industry, plants, development, quality, efficient, management, operation, developed, based, solutions, manufacturing, industrial, presented, approach, technologies, integration, operational, production, application, information, applications, advanced, costs, cost, mill, environmental, improvement, processes, high	Technology for Energy Efficiency
02	steel, casting, inclusions, continuous, carbon, inclusion, process, surface, low, slab, analysis, steelmaking, quality, mold, cast, grades, rolling, steels, size, high, clogging, thin, number, formation, nozzle, tundish, treatment, production, ladle, samples, aluminum, work, method, content, solidification, investigated, addition, nitrogen, defects, wire	Continuous Casting in Steelmaking
03	steel, industry, production, global, environmental, energy, technologies, market, products, challenges, development, years, efficiency, countries, companies, world, recent, european, sector, policy, processes, sustainable, sustainability, major, innovation, industries, barriers, business, materials, important, international, role, issues, prices, efforts, demand, activities, increasing, product, project	Global Steel Industry
04	electric, furnace, eaf, arc, scrap, energy, steelmaking, process, furnaces, efficiency, melting, electrical, steel, hot, productivity, power, oxygen, increase, input, production, plant, metal, route, consumption, operation, reduced, operating, dri, direct, continuous, materials, chemical, charge, processes, increased, conditions, developed, operations, cost, time	Electric Arc Furnace in Steelmaking → Electrification
05	heat, energy, system, power, recovery, waste, water, efficiency, exergy, steam, cooling, thermal, heating, air, plant, systems, generation, utilization, storage, temperature, high, proposed, loss, pressure, capacity, gas, based, plants, analysis, electricity, cycle, turbine, transfer, technologies, potential, load, case, low, performance, recovered	Heat waste recovery to system efficiency → CDQ
06	co2, hydrogen, gas, steel, emissions, process, carbon, production, capture, reduction, electricity, steelmaking, iron, plant, natural, direct, processes, fuel, storage, cost, fossil, gases, integrated, emission, dioxide, renewable, reduce, system, industry, compared, produced, fuels, sources, mill, energy, based, reducing, plants, technology, methanol	Carbon Capture and Storage, Hydrogen, Electricity, and Direct Reduction iron in Steelmaking
07	steel, industry, development, iron, carbon, technology, china, low-carbon, process, green, technologies, production, future, enterprises, china's, key, emission, application, current, structure, important, high, based, system, metallurgical, reduction, neutrality, emissions, technical, progress, utilization, energy, analysed, energy-saving, achieve, status, china, transformation, chinese	Low Carbon Development in Iron Steel Industry: Technology
08	emission, emissions, reduction, carbon, co2, industry, steel, iron, china, air, scenario, total, pollution, coal, china's, industrial, policy, dioxide, tons, so2, million, policies, pollutants, measures, control, nox, industries, pm2.5, model, production, tax, pollutant, reduce, 2030, 2020, scenarios, sources, contribution, output, region	CO2 Emission Reduction in Steel Industry
09	steel, steels, properties, low, rolling, strength, mechanical, high, hot, microstructure, carbon, grain, processing, ferrite, plate, toughness, cooling, temperature, rolled, materials, test, production, developed, plates, material, process, strip, structure, performance, phase, design, corrosion, applications, improved, yield, cold, tensile, strain, higher, composition	Properties of Steel
10	refractory, carbon, stainless, thermal, refractories, jet, high, resistance, steel, ladle, lining, chromium, materials, lance, bricks, supersonic, application, oxygen, graphite, low, conventional, furnace, properties, aod, depth, electrode, mgo-c, corrosion, coherent, layer, development, bath, steelmaking, brick, refining, rate, injection, material, compared, effect	Refractory
11	slag, waste, materials, recycling, material, cement, production, process, raw, steel, recovery, solid, dust, concrete, co2, products, mineral, environmental, carbonation, resource, utilization, wastes, slags, produced, reduction, industrial, product, treatment, zinc, high, metals, water, calcium, potential, emission, resources, processing, mineralization, methods, construction	Waste Materials Recycling
12	energy, consumption, steel, efficiency, production, iron, saving, industry, process, conservation, analysis, material, flow, potential, system, reduce, total, savings, data, intensity, management, making, processes, improvement, integrated, specific, plant, based, measures, ton, intensive, energy-saving, industrial, factors, main, plants, model, industries, found, improving	Energy Consumption, Efficiency and Conservation in Iron Steel Production
13	process, carbon, steel, reaction, rate, decarburization, liquid, content, gas, oxygen, phase, oxide, oxidation, mass, molten, steelmaking, low, thermodynamic, investigated, vacuum, formation, removal, interface, copper, model, solid, high, nitrogen, processes, thermodynamics, found, flow, sulphur, experimental, kinetics, contents, based, pressure, layer, time.	Decaburization Process
14	environmental, efficiency, energy, economic, analysis, impact, effect, industrial, life, factors, cycle, iron, assessment, data, performance, industry, impacts, indicators, effects, steel, change, growth, evaluation, method, benefits, model, structure, level, index, symbiosis, studies, analyze, decoupling, intensity, china's, significant, factor, based, technical, evaluate	Energy Efficiency: Economic, Life Cycle Analysis
15	iron, ore, coal, biomass, reduction, coke, strength, reaction, pellets, temperature, ratio, properties, carbon, powder, process, content, high, higher, pellet, reactivity, charcoal, gasification, time, addition, size, effect, increased, particle, compressive, ores, composite, carbonization, ironmaking, increase, pyrolysis, conditions, ichb, hematite, behavior, performance	Iron Ore Reduction using biomass.
16	steel, emissions, production, technologies, industry, co2, sector, potential, global, climate, industrial, mitigation, ghg, carbon, demand, 2050, energy, scenario, future, decarbonization, reduction, scenarios, sectors, iron, material, electricity, cement, primary, industries, options, current, supply, costs, greenhouse, change, technology, targets, analysis, strategies, ccs	Emission Reduction in Steel Production: Technology, Mitigation, Scenario, Decarbonization, Electricity, Strategy
17	model, production, optimization, method, proposed, based, data, process, algorithm, optimal, problem, energy, scheduling, steel, models, system, prediction, cost, network, time, simulation, parameters, mathematical, programming, established, efficiency, operation, dynamic, developed, plant, constraints, control, carbon, linear, approach, actual, improve, objective, strategy, learning	Production Models Optimization
18	blast, furnace, gas, reduction, process, coke, ironmaking, emission, sintering, injection, rate, sinter, reducing, carbon, high, flue, burden, utilization, operation, oxygen, iron, fuel, reduce, coal, top, oven, co2, hot, temperature, low, consumption, reduced, saving, zone, energy, ratio, technology, shaft, hydrogen, smelting	Blast Furnace Gas Reduction
19	slag, steel, oxygen, converter, content, metal, molten, steelmaking, process, temperature, ladle, bof, blowing, smelting, ratio, high, composition, hot, low, refining, rate, production, lime, manganese, increases, dephosphorization, iron, increasing, carbon, limestone, point, increase, phosphorus, main, effect, metallurgical, control, basicity, cao, end	Steel Slag Oxygen Converter
20	temperature, heat, combustion, model, flow, furnace, transfer, rate, simulation, gas, conditions, surface, heating, effect, bed, distribution, spray, thermal, reheating, process, experimental, cooling, efficiency, due, air, mass, flux, flame, fluid, developed, analysis, particles, numerical, time, increases, size, parameters, method, high, oxygen	Heat Temperature Combustion Model

An interview with a business actor in the steel industry was presented that Indonesia crude steel production was about 4.8 million tonnes, requiring energy of around 20.3 Gj/tcs or 5623 kWh/tcs. The share of energy cost to the total production cost is about 20–25%. The average level of emissions is approximately 1.9 CO2/tcs. In 2035, the steel demand will peak, and demand growth is expected to decrease from 5% to about 1.1%. It seems that between 1960 and 2010, the index of energy consumption per tonne of crude steel production substantially dropped from 100 to about 40, which means about 60 points improvement in energy efficiency whereas, for the last seven years, it has been constant at about 40.

Krakatau Posco is a joint venture company between leading global steelmakers POSCO and PT Krakatau Steel Indonesia. In term of low carbon development, Krakatau Steel has implemented several technologies to improve energy efficiency in the production processes i.e., partial oxidation in direct reduction iron (DRI) to save natural gas consumption, including advanced heating systems such as neuro furnace controller, walking-beam furnace, high-efficiency burners, and pulverized coal injection (PCI) system whereas the adoption of hydrogen-based technology is regarded as future potentials. Krakatau Posco has also adopted best-practice technology that has been developed in the Gwangyang plant in the Republic of Korea. In pursuing an energy-efficient technology and the low-carbon green development of the domestic steel industry, POSCO adopted CDQ by using nitrogen instead of water. CDQ using nitrogen provides better heat recovery and energy efficiency compared to the water quenching method. The hot coke is cooled down rapidly using nitrogen, allowing for efficient heat exchange and energy recovery. CDQ is a widely used method for recovering waste heat in the steel industry which can be used to produce steam and electricity or pre-heating cooking coal. This technology can reduce CO2 emissions and thermal energy loss. This technology can also improve coke quality and reduce coke consumption in the blast furnace. In addition, CDQ can decrease non-cooking coal and reduce costs. However, the application of CDQ needs substantial investment. CDQ is the second most expensive technology whereas the lowest in penetration rate among other technologies in reducing emission in iron and steel industry [47]. If the electricity produced from CDQ can be sold at a competitive price, it may result in a high internal rate of return (IRR). Hence, promoting CDQ into the carbon market is essential to gain its full benefit. Nonetheless, achieving this goal necessitates collaborative endeavours aimed at standardizing CO2 emission accounting methodologies and instituting a carbon trading system [55].

By design, steel production has great potential to generate energy use for other processes. Because the steelmaking process produces energy and can be treated as captive power, then power trading between steel and state-owned electricity companies can be promoted under a win-win solution. In this case, the energy auditor plays an essential role in conducting an energy and/or emission audit in the company and proposes an improvement by promoting new investment that benefits from carbon pricing mechanisms. By improving efficient use of energy, steel companies will be able to increase their competitiveness. Then, by re-using waste of steam and heat into an energy source, it will provide benefits for the company. However, the investment costs required are quite expensive, such as for CDQ technology. In this case, the government can act as an off taker who buys excess power, and this will encourage companies to use increasingly environmentally friendly technology.

## 4 Conclusion and policy implications

Indonesia has great potential to achieve a low-carbon economy. The review of two sectors, namely electricity and steel showed that the transition toward low carbon is underway. The development of renewable energy in the energy system is prospective. Solar PV, pumped storage hydropower, geothermal, and energy from waste have been applied to the plant's energy system. The development of renewable energy potential requires support in the electricity system, starting from transmission, distribution, and metering technology. Thus, technological disruption will provide optimal benefits if appropriate transformation occurs in the supporting system. Likewise, disruption occurs on the demand side. More and more consumers, especially at the company and household level, want to buy electricity only from renewable energy sources. The greater the change on the demand side, the greater the economic feasibility of driving technological change.

Furthermore, the industrial sector has the remaining challenge. Promoting energy efficiency to reduce energy costs has become a strategic and dominant choice. Battery technology advancement can expand renewable energy penetration and disrupt the energy system. Smart grids become the key driver toward a new paradigm in the transition to renewable energy and promoting energy efficiency in the distribution and use of electricity. On the other hand, the functionalities of the smart system will not be optimal without the availability of an energy storage system whereby the battery plays a key role. Therefore, the national long-term energy strategy should put these two into the main components of development.

The country needs to develop a strategic position for this transformation in the energy sector. One of the key principles of strategic positioning requires countries to make trade-offs in competing activities in the short run, while developing innovation. Therefore, Indonesia needs to gradually give up high carbon energy content for low carbon energy sources. In the electricity sector, smart grids, renewable energy, especially wind and solar PV, clean coal power generation such as coal gasification, CCUS, ultra-supercritical technology, fluidized bed combustion and IGCC, and gasification of the existing diesel plant as well as biomass co-firing for the existing coal fired power plants, are critical. We found that the choice of technology depends on various factors, including the power plant's size, energy requirements, grid compatibility, cost considerations, environmental regulations, location, and demand condition. In the energy-intensive industries such as steel, the energy efficiency targets at the industrial level are important. Technology choices will also be ‘firm specific’, especially the technology platform of the parent company. This condition means that systematic literature reviews have not been able to identify it. This weakness can be overcome by conducting case studies at the company level.

The global technological disruption provides the dual track approaches of technology development and innovations, namely energy efficiency and renewable energy. Indonesia can gain many benefits from the global technological advancement. However, Indonesia needs to optimize the transition by promoting changes in system support of power sector and changing consumer behavior toward green civilization. In terms of policy, the Indonesian government needs to continue to encourage sustainable technology development and innovations. Because disruption process can be developed in a specific company context, government need to facilitate this by providing various incentives to the companies by such as price policies, terms of contracts, and infrastructure support. This process will promote the nation's competitiveness advantage.

## Data availability

Data will be made available on request.

## CRediT authorship contribution statement

**Maxensius Tri Sambodo:** Writing – review & editing, Writing – original draft, Supervision, Resources, Methodology, Investigation, Funding acquisition, Formal analysis, Data curation, Conceptualization. **Mesnan Silalahi:** Writing – review & editing, Writing – original draft, Visualization, Software, Methodology, Investigation, Formal analysis, Data curation, Conceptualization. **Nur Firdaus:** Writing – review & editing, Resources, Data curation.

## Declaration of competing interest

The authors declare that they have no known competing financial interests or personal relationships that could have appeared to influence the work reported in this paper.

## References

[1] OECD (2010).

[2] Schumpeter J.A. (1934).

[3] Christensen C.M. (1997).

[4] Besanko D., Dranove D., Shanley M., Schaefer S. (2013).

[5] Christensen C.M., Raynor M.E. (2003).

[6] Danneels E. (2004). Disruptive technology reconsidered: a critique and research agenda. J. Prod. Innovat. Manag..

[7] Wilson C., Pettifor H., Cassar E., Kerr L., Wilson M. (2019). The potential contribution of disruptive low-carbon innovations to 1.5 C climate mitigation. Energy Efficiency.

[8] Cornell University INSEAD and the World Intellectual Property Organization (2018).

[9] Tayal D. (2016). Disruptive forces on the electricity industry: a changing landscape for utilities. Electr. J..

[10] Gielen D., Boshell F., Saygin D., Bazilian M.D., Wagner N., Gorini R. (2019). The role of renewable energy in the global energy transformation. Energy Strategy Rev..

[11] World Energy Council [WEC] (2017).

[12] Rogers M. (2019). https://www.mckinsey.com/capabilities/sustainability/our-insights/sustainability-blog/these-9-technological-innovations-will-shape-the-sustainability-agenda-in-2019.

[13] Andoni M., Robu V., Flynn D., Abram S., Geach D., Jenkins D., McCallum P., Peacock A. (2019). Blockchain technology in the energy sector: a systematic review of challenges and opportunities. Renew. Sustain. Energy Rev..

[14] (2022). NDC Indonesia.

[15] Lowes R., Woodman B. (2020). Disruptive and uncertain: policy makers' perceptions on UK heat decarbonisation. Energy Pol..

[16] IRENA (2022). World Energy Transitions Outlook.

[17] Gough D., Oliver S., Thomas J., Gough D., Oliver S., Thomas J. (2012). An Introduction to Systematic Reviews.

[18] Callon M., Law J., Rip A., Callon M., Law J., Rip A. (1986). Mapping the Dynamics of Science and Technology: Sociology of Science in the Real World.

[19] Blei D.M., Ng A.Y., Jordan M.I. (2003). Latent dirichlet allocation. J. Mach. Learn. Res..

[20] Kusumastuti R., Silalahi M., Asmara A.Y., Hardiyati R., Juwono V. (2022). Finding the context indigenous innovation in village enterprise knowledge structure: a topic modelling. Journal of Innovation and Entrepreneurship.

[21] Timulak L. (2014).

[22] International Energy Agency [IEA] (2017).

[23] International Energy Agency [IEA] (2017).

[24] International Energy Agency [IEA] (2011).

[25] Zhang C., Zhai H., Cao L., Li X., Cheng F., Peng L., Wang .X. (2022). Understanding the complexity of existing fossil fuel power plant decarbonization. iScience.

[26] Paltsev S., Morris J., Kheshgi H., Herzog H. (2021). Hard-to-Abate Sectors: the role of industrial carbon capture and storage (CCS) in emission mitigation. Appl. Energy.

[27] Schittkowski J., Ruland H., Laudenschleger D., Girod K., Kähler K., Kaluza S., Schlögl .R. (2018). Methanol synthesis from steel mill exhaust gases: challenges for the industrial Cu/ZnO/Al2O3 catalyst. Chem. Ing. Tech..

[28] Kartal F., Özveren U. (2023). Energy and exergy analysis of entrained bed gasifier/GT/Kalina cycle model for CO2 co-gasification of waste tyre and biochar. Fuel.

[29] Stanger R., Wall T., Spörl R., Paneru M., Grathwohl S., Weidmann M., Santos .S. (2015). Oxyfuel combustion for CO2 capture in power plants. Int. J. Greenh. Gas Control.

[30] Hou Y., Feng Q., Wang C., Gao X., Che D. (2023). Numerical simulation on co-firing ultra-low volatile carbon-based fuels with bituminous coal under oxy-fuel condition. Fuel.

[31] Arifin Z. (2019). 2019 IEEE Technology & Engineering Management Conference (TEMSCON).

[32] Lovins A. (2013).

[33] International Energy Agency [IEA] (2023).

[34] Neffati O.S., Sengan S., Thangavelu K.D., Kumar S.D., Setiawan R., Elangovan M., Velayutham .P. (2021). Migrating from traditional grid to smart grid in smart cities promoted in developing country. Sustain. Energy Technol. Assessments.

[35] PT PLN (Persero) (2023). https://connect-gateway.pln.co.id/magazine/magazine/articles/detail/pln-segera-gunakan-ami-tahun-ini-meteran-canggih-yang-bikin-pelanggan-bisa-monitor-penggunaan-listrik-secara-realtime.

[36] Susanto D.A., Louhenapessy B.B. (2014). Ketersediaan Standar dalam Mendukung Penerapan Sistem Smart Grid di Indonesia (Availability of Standard in Supporting Implementation of Smart Grid in Indonesia). J. Stand..

[37] Julianto K., Nugraha D.W., Dodu A.Y.E. (2014). Evaluasi penggunaan SCADA pada keandalan sistem distribusi PT. PLN (Persero) area Palu. Jurnal Mektrik.

[38] Alves Dias P., Blagoeva D., Pavel C., Arvanitidis N. (2018).

[39] IEA, Hydropower (2021).

[40] Sambodo M.T., Yuliana C.I., Hidayat S., Novandra R., Handoyo F.W., Farandy A.R., Inayah I., Yuniarti P.I. (2022 Apr 19). Breaking barriers to low-carbon development in Indonesia: deployment of renewable energy. Heliyon.

[41] Chen C., Yang A. (2021). Power-to-methanol: the role of process flexibility in the integration of variable renewable energy into chemical production. Energy Convers. Manag..

[42] Bermúdez J.M., Arenillas A., Luque R., Menéndez J.A. (2013). An overview of novel technologies to valorise coke oven gas surplus. Fuel Process. Technol..

[43] Ren M., Lu P., Liu X., Hossain M.S., Fang Y., Hanaoka T., Dai .H. (2021). Decarbonizing China's iron and steel industry from the supply and demand sides for carbon neutrality. Appl. Energy.

[44] Zhi-feng C.U.I., An-jun X.U., Fang-qin S.H. (2022). Low-carbon development strategy analysis of the domestic and foreign steel industry. 工程科学学报.

[45] Na H., Sun J., Qiu Z., Yuan Y., Du T. (2022). Optimization of energy efficiency, energy consumption and CO2 emission in typical iron and steel manufacturing process. Energy.

[46] Flores-Granobles M., Saeys M. (2020). Minimizing CO2 emissions with renewable energy: a comparative study of emerging technologies in the steel industry. Energy Environ. Sci..

[47] Liu X., Peng R., Bai C., Chi Y., Li H., Guo P. (2022). Technological roadmap towards optimal decarbonization development of China's iron and steel industry. Sci. Total Environ..

[48] Branca T.A., Colla V., Algermissen D., Granbom H., Martini U., Morillon A., Rosendahl S. (2020). Reuse and recycling of by-products in the steel sector: recent achievements paving the way to circular economy and industrial symbiosis in Europe. Metals.

[49] Xue Y.L., Zhang J., Liu Y., Chen Y., Sun J., Jiang H.Q., Cao .D. (2022). Roadmap of coal control and carbon reduction in the steel industry under the carbon peak and neutralization target. Huan Jing ke Xue= Huanjing Kexue.

[50] Devlin A., Yang A. (2022). Regional supply chains for decarbonising steel: energy efficiency and green premium mitigation. Energy Convers. Manag..

[51] Demeri M.Y. (2013).

[52] World Steel Association [WSA] (2014).

[53] Fan Z., Friedmann S.J. (2021). Low-carbon production of iron and steel: technology options, economic assessment, and policy. Joule.

[54] Shahabuddin M., Brooks G., Rhamdhani M.A. (2023). Decarbonisation and hydrogen integration of steel industries: recent development, challenges and technoeconomic analysis. J. Clean. Prod..

[55] Zhang J., Shen J., Xu L., Zhang Q. (2023). The CO2 emission reduction path towards carbon neutrality in the Chinese steel industry: a review. Environ. Impact Assess. Rev..

